# Attitudes and perceptions towards developing a health educational video to enhance optimal uptake of malaria preventive therapy among pregnant women in Uganda: a qualitative study involving pregnant women, health workers, and Ministry of health officials

**DOI:** 10.1186/s12913-024-10944-x

**Published:** 2024-04-18

**Authors:** Rita Nakalega, Ruth Nabisere-Arinaitwe, Nelson Mukiza, Cynthia Ndikuno Kuteesa, Denis Mawanda, Paul Natureeba, Ronnie Kasirye, Clemensia Nakabiito, Jane Nabakooza, Emmie Mulumba, Josephine Nabukeera, Joseph Ggita, Abel Kakuru, Lynn Atuyambe, Philippa Musoke, Mary Glenn Fowler, Zubair Lukyamuzi

**Affiliations:** 1 Makerere University-Johns Hopkins University (MU-JHU) Research Collaboration, Kampala, Uganda; 2 Infectious Diseases Institute, Makerere University, Kampala, Uganda; 3 RineCynth Advisory, Kampala, Uganda; 4 Ministry of Health, Kampala, Uganda; 5 Infectious Diseases Research Collaboration, Kampala, Uganda; 6 Makerere University, Kampala, Uganda; 7 John Hopkins University, Baltimore, USA

**Keywords:** Malaria, Preventive therapy, Pregnancy, Educational video, Qualitative study

## Abstract

**Background:**

Malaria in pregnancy remains a major global public health problem. Intermittent prophylaxis treatment of malaria in pregnancy with Sulphadoxine-pyrimethamine and co-trimoxazole is efficacious for prevention of malaria in pregnancy HIV negative and positive women, respectively. However, uptake of the recommended doses of therapies has remained suboptimal in Uganda, majorly due to inadequate knowledge among pregnant women. Therefore, this study aimed to explore attitudes and perceptions towards developing an educational video for malaria preventive therapy.

**Methods:**

We conducted an exploratory study with qualitative methods among pregnant women attending antenatal care at Kisenyi Health Center IV (KHCIV), health workers from KHCIV, and officials from the Ministry of Health. The study was conducted at KHCIV from October 2022 to March 2023. Focus group discussions (FGD) were conducted among purposively selected pregnant women and key informant interviews (KII) among health workers and Ministry of Health officials. Data were analyzed using inductive and deductive thematic methods in atlas ti.8.

**Results:**

A total of five FGDs comprising of 7–10 pregnant women were conducted; and KIIs were conducted among four mid-wives, two obstetricians, and two Ministry of Health officials. Generally, all respondents mentioned a need for interventions to improve malaria preventive knowledge among pregnant women; were positive about developing an educative video for malaria preventive therapy in pregnancy; and suggested a short, concise, and edutaining video focusing both the benefits of taking and risks of not taking malaria preventive therapy. They proposed that women may be encouraged to view the video as soon as they conceive and throughout the pregnancy. It also was suggested that the video may be viewed on television sets in maternal and reproductive health clinics and homes, and on smart phones.

**Conclusion:**

Pregnant women, health workers, and Ministry of Health officials were positive about the development of a short edutaining video on malaria preventive therapy that focuses on both benefits of taking and risks of not taking the malaria preventive therapy in pregnancy. This information guided the video development and therefore, in the development of health educative videos, client and stakeholder inputs may always be solicited.

**Supplementary Information:**

The online version contains supplementary material available at 10.1186/s12913-024-10944-x.

## Introduction

Malaria in pregnancy remains a major public health concern worldwide; more than 50 million pregnant women live in malaria-endemic areas with 88% in sub-Saharan Africa (SSA) [1, 2]. Malaria in pregnancy is associated with undesirable fetal and maternal outcomes including miscarriage, low birth weight, stillbirth, maternal morbidity, and mortality; moreover, malaria is the leading cause of preventable fetal and maternal deaths [3–5]. These undesirable fetal and maternal outcomes are worsened by coinfection with other diseases particularly HIV [6–8]; and Uganda remains one of the most affected countries by both HIV and malaria [9–12]. The country is malaria endemic with the prevalence malaria in pregnancy ranging from 8.9 to 51.1% [13–15], and HIV prevalence of 8% among women of reproductive age [16].

Intermittent prophylaxis treatment of malaria in pregnancy with Sulphadoxine-pyrimethamine (IPTp-SP) and co-trimoxazole (CTx) is efficacious for prevention of malaria in pregnancy in pregnant women without HIV and those with HIV, respectively [17, 18]. These medications have shown to reduce malaria parasitemia in pregnancy by approximately 80% [18, 19]. In HIV negative women, IPTp-SP is initiated after the 1st trimester and taken on a monthly basis throughout the pregnancy [20, 21] whereas, CTx is taken on a daily basis throughout the pregnancy for women living with HIV [11, 22]. Although antenatal care (ANC) attendance in Uganda had increased to 95%, uptake of the recommended doses of IPTp-SP and CTx had remained suboptimal especially in the urban areas [3, 23]. In the country’s capital, Kampala, optimal uptake of IPTp-SP was as low as 39% in 2018 [3].

Multi-sectoral factors contribute to sub-optimal uptake of malaria preventive therapy in pregnancy. However, lack of education, ignorance, and inadequate awareness about malaria preventive therapy among pregnant women and health workers remain the major factors [3, 24–27]. This is partly due to persistent low ratio of health workers to patients in Low Resource Settings (LRS) [28]; resulting in inadequate sensitization and education of women on malaria preventive therapy during ANC visits. Hence, a critical need for interventions to improve knowledge and uptake of malaria preventive therapy during pregnancy.

Brief video-based educational interventions are promising approaches in improving health related knowledge among the target populations [29]. These videos are highly cost-effective [29, 30], have a strong track record in improving knowledge, and fostering behavior change in various health related fields including HIV [29, 31–33]. However, the responses and reactions to these interventions among stakeholders in malaria prevention during pregnancy remain unknown.

In this study, we recorded client and stakeholder opinions, attitudes, and perceptions, to the development of an educational video to improve knowledge and uptake of malaria preventive therapy in pregnancy.

## Methods

### Study design and setting

This was a sub-study of a larger two phased sequential cross-sectional study that used a client- centered and stakeholder consultative approach to develop an educational video on malaria preventive therapy in pregnancy. The primary study had two phases; the first phase aimed to solicit inputs from clients and stakeholders in the development of an educative video for improving knowledge of malaria preventive therapy among pregnant women; and the second phase aimed to assess the feasibility and acceptability of the developed video. Therefore, the current study describes the first phase of the primary study. This phenomenological qualitative study was conducted at Kisenyi Health Center IV (KHCIV) from October 2022 to March 2023. KHCIV is a public health facility in Kampala Central division, administered by Kampala Capital City Authority (KCCA). In Uganda, a HCIV provides general health services plus minor surgeries [34, 35]. The facility has a catchment area of ≈ 2,000,000 people from Kampala and suburbs, and serves over 200 persons per day [36]. It offers free HIV/TB Care services, ANC services, and other services. Daily, the facility had ANC attendance of about 100 women and conducted about 20 births.

### Study population

The study population included pregnant women at any gestation age attending ANC at KHCIV during the study period and key informants. The key informants were obstetricians and midwives from maternal and child health department at KHCIV, and Ministry of Health (MoH) officials from the malaria control department.

### Study outcomes

The study outcomes were opinions, attitudes, and perceptions of the study participants about the development of an educative video on malaria preventive therapy in pregnancy.

### Sample size and sampling procedures

Fifty pregnant women attending ANC at KHCIV were purposively selected and enrolled in the study. Women were stratified by age and HIV status to undergo focus group discussions (FGD). A total of five FGDs each comprising of 7–10 women were conducted among HIV negative women aged < 18years, 18-24years, and 25-49years; and women living with HIV aged 18-24years, and 25-49years. Due to the limited number of women living with HIV aged < 18 years during the study period, we were unable to get enough women for a FGD for this age group. We, therefore, instead conducted an in-depth interview (IDI) with a teenager living with HIV. We also conducted key informant interviews (KII) among obstetricians, midwives, and Ministry of Health (MoH) officials. Focus group discussions, IDI, and KIIs were conducted until when saturation was reached [37] in each group.

### Data collection procedures

Pregnant women attending ANC at KHCIV were purposively selected and informed about the study, and those who were interested were consented to participate. Women were selected from the ANC clinic daily and scheduled for FGDs. Demographics and clinical information were obtained from all participants using a questionnaire before participating in the FGDs. The FGDs were based on open discussion, and each lasted typically for about two hours in duration. Using an FGD guide (Appendix 1), the discussions were conducted by a trained social scientist (facilitator) in a calm place where conversations could not be overhead. Similarly, the IDI for a teenager living with HIV and KIIs for key informants were conducted in a calm place where conversations could not overhead. The interviews lasted between 15 and 30 min and were conducted face to face using an IDI or KII guide. The FGDs, IDI, and KIIs were conducted in respondents’ preferred language which were Luganda or English. All the interviews were audiotaped and transcribed by a professional. The study documents such as structured questionnaire, FGD/IDI guides, and consents were translated from English to Luganda, the local language used. These were then back translated to English; and we then compared the new translation with the original text and reconciled any meaningful differences between the two to ensure that the translations were accurate.

### Data management

Audio recordings were transcribed verbatim directly into English by the study team within one week of collection. Quality checks were performed for each transcript, with corrections and revisions made to identified errors.

### Data analysis

Using Stata version 17 (StataCorp, College Station, TX, USA), descriptive continuous variables, such as age, were summarized using mean and standard deviation (SD) while categorical variables were summarized using frequencies and proportions.

### Qualitative data analysis

Using an inductive and deductive content analytic approach, recorded data were transcribed and analyzed by the study team supervised by a qualitative research expert, using Atlas t.8 software. The initial review was done during debriefing process to give a baseline understanding of the data and constant comparison process continued to identify new information until there was no redundancy in the themes and this was part of the steps during inductive approach.The Consolidated Criteria for Reporting Qualitative Studies checklist was used to report study findings [38]. During the inductive analysis, open coding was carried out to identify specific portions of text corresponding to information required for the video development. Provisional labels were defined and illustrated to become codes, which were assembled into a codebook. Data was coded by two coders (social scientist and corresponding author). After development of the initial codebook, the codebook was reviewed for consistency of text segmentation and code application with continued inter-coder agreement. The coders reached a consensus and grouped identified codes into grouped sub-category, category and then into subthemes and themes. Coded themes from all data were compared to obtain generalized themes after removing inconsistent codes. The choice of thematic headings was guided by both the core concepts emerging from the data [39] and by theoretical concepts of the health belief model (HBM) [40]. During the second phase of analysis, specific topics were designated as core categories; axial coding and constant comparison to explore the relationships between the discussion of sensitive data and contextual situation [41]. Findings and interpretations of the data were discussed until there was group consensus on the dominant themes and meanings contained in the data.

### Ethical approval

Approval to conduct this study was obtained from the Makerere University School of Medicine Research Ethics Committee (Mak-SOMREC-2021-279) and Uganda National Council for Science and Technology (NS384ES). Administrative clearance was obtained from the Director of Health Services at KCCA and from KHCIV administration. Written informed consent was obtained from all participants; confidentiality and anonymity were strictly observed. Informed consent for illiterate participants was obtained in the presence of an impartial witness (guardian or other literate person not part of study team). The procedure of obtaining informed consent from illiterate participants was approved by the Makerere University School of Medicine Research Ethics Committee IRB. All methods were performed in accordance with the relevant guidelines and regulations of good clinical practice and human subject protection of ICH-6.

## Results

A total of 50 women were enrolled for FGDs and IDI. Eight key informants were also enrolled for key informant interviews. These included two obstetricians, four mid-wives, and two Ministry of Health officials. The mean age of the enrolled women was 23 years (SD ± 6 years); the majority, 26 (53.1%) had completed primary level of education and the median gestation age was 24 weeks with interquartile range (IQR) of 20–32 weeks. The majority 36 (73.5%) were currently living with their partners and most 41 (83.7%) were receiving financial support from their partner. The majority 27(55.1%) used 1–2 h to travel to the ANC facility (KHCIV) and most 26 (53.1%) had had more than two ANC visits, and the average number of ANC visits was 3 as shown in Table 1.

**Table 1:** Socio-demographic characteristics of the pregnant women enrolled in the study

Characteristics		Frequency	Percentage (%)
Age	Mean (SD)	23 (6) years	
District of residence			
	Urban	34	69.4
	Semi-Urban	15	30.6
Education Level			
	Primary not completed	12	24.5
	Primary completed	26	53.1
	S4 completed	8	16.3
	S6 Completed	3	6.1
Currently living with partner			
	Yes	36	73.5
	No	13	26.5
Financial Support from partner			
	Yes	41	83.7
	No	8	16.3
Time taken to reach the health facility			
	Less than 1 h	21	42.9
	1–2 h	27	55.1
	More than 2 h	1	2.0
ANC visits (In days)			
	≤ 2 ANC visits	23	46.9
	> 2 ANC visits	26	53.1
Gestation (In weeks)			
	≤ 24 weeks	27	57.5
	> 24 weeks	20	42.5

To thematically categorize opinions, perceptions, and attitudes of respondents, we used the HBM [40] and Gain/Loss messaging Framework [42]. The HBM postulates that cues to action and presence of an enabling environment are among the vital constructs in the uptake of a particular behavior [40]. The Gain/Loss framework describes the packaging of a message focusing on the positive benefits of doing something or the risk of not doing something [42] as shown in Table 2.

**Table 2:** Examples of themes obtained from respondents regarding cues to action, and message packaging according to the HBM and Gain/Loss framework

Construct	Quote	Code	Category	Theme
Needed actions and strategies to improve knowledge of malaria preventive therapy in pregnancy	*…Yes, educate them on television, radio, and social media. Many women have these means*	Increasing awareness and sensitization on malaria preventive therapy	Providing regular health education	Improving knowledge among women regarding malaria preventive therapy
Packaging and Framing of the message of malaria preventive therapy in the video.	*“the video should have well packaged key messages. You know people’s concentration, especially for the adults is very short. So, the video should be short. And should focus on both benefits and risks but more focus on benefits of taking malaria preventive therapy*.	Relevancy, length, and focus of the video	Contents of an educative video for malaria preventive therapy.	Enhancing messaging of malaria preventive therapy in a video-based intervention.
Delivery of the message of malaria preventive therapy in the video.	*“It’s a good innovation because, you know how people, especially those in reproductive age want to watch videos. You realize that someone can sit somewhere pick up their phone and watch the videos that they have on their phones. And if there is a TV, for example in the clinic or at home, people will watch it*	Delivering the video-based information for malaria preventive therapy	Appropriate means of delivering the video-based information for malaria preventive therapy	Enhancing access to malaria preventive therapy information among pregnant women.

Four broad themes emerged from the data to describe the use of video-based intervention to educate women about malaria preventive therapy in pregnancy. These themes were: needed actions and strategies to improve knowledge of malaria preventive therapy in pregnancy; positive attitudes towards the use of the video-based intervention to educate women on malaria preventive therapy in pregnancy; Packaging and Framing of the message of malaria preventive therapy in the video; and delivery of the message of malaria preventive therapy in the video.

### Strategies and actions needed to improve knowledge of malaria preventive therapy in pregnancy

Respondents acknowledged the need for strategies to improve knowledge about malaria preventive therapy in pregnancy; and they mentioned various strategies to improve knowledge and uptake of IPTp-SP and co-trimoxazole during pregnancy. The mentioned strategies included engagement of male sexual partners or treatment supporters and providing regular health education talks.

Regarding male partner and treatment supporter involvement, *r*espondents mentioned that the involvement males and treatment supporters could help to enhance women’s understanding of the purpose and benefits of taking malaria preventive therapy. The male partners and treatment supporters can educate further the women and can remind them to swallow the medicines in case the women forget.

*“We always counsel women to bring their partners or come with a treatment supporter. This helps because if a woman has not understood the message or information given, the treatment supporter or partner can understand and explain or remind her.“*, midwife III.

For continuous educational talks, respondents mentioned that efforts should be made to provide comprehensive and repeated health education talks during ANC to minimize malaria preventive therapy knowledge deficits among women. Respondents suggested various strategies including education and sensitization of women during ANC visits and the use of communication materials (IEC), village health team (VHT) system, and media-based education such as televisions, radios, and social media.

*“Like I told you earlier, providing women with information regarding the use of malaria preventive therapy including how to minimize the associated side effects will improve uptake. Women can be organized in small groups during ANC in addition to one-on-one sessions. We should also ensure that the information given reaches the sexual partners and treatment supporters”*, Obstetrician II.

*“Women need to be adequately educated on why they should take these drugs to improve uptake and adherence”.* FGD lady aged _15–17 yrs.

Demonstrations and illustrations were also encouraged during education talks.

*“Yes, because with the flip chart, you can easily illustrate the importance of taking malaria preventive therapy. You can also have brochures which can show various actions on different pages. For example, you can show a happy woman taking IPTp-SP or co-trimoxazole on the first page, and on next page you show a healthy born baby”*, midwife. III.

*“…like there various apps on phones, they can also develop for us a malaria prevention app to teach women about malaria. I can have that App on my phone and can learn the schedule for taking malaria preventive therapy”.* FGD woman 25-49yrs.

### Positive attitudes to developing and designing an educative video for malaria preventive therapy

All respondents agreed that an educative video was one of the best interventions that can improve knowledge and uptake of malaria preventive therapy in pregnancy. This is because videos can be easily understood and always attract and capture women’s attention.

*“The video can be good and more receptive in educating women because many women don’t know why should take these drugs. We not only need to have health workers acting in the video but also pregnant women. We can have women giving success stories”.* Obstetrician I.

“*By viewing, we are entertained but also being educated. Yah, I think it is a good innovation”. MoH official I*.

*“It’s a good innovation because, you know how people, especially those in reproductive age want to watch videos. MoH official II.*

### Packaging and Framing of the message of malaria preventive therapy in the video

Respondents mentioned how to package and provide (message) information in the video.

#### Packaging information in the video

Regarding packaging, respondents suggested various ways of packaging the information in the video. They mentioned that the video should be short and concise, focused, engaging or edutaining, and be acted by real human beings.

#### Short, concise, and focused video

Respondents mentioned that for the video to be effective and efficient, it should be short, concise, and focused. This is because some people don’t concentrate for long.

*“The video should have well packaged key information. You know people’s concentration, especially for adults, is very short. So, the video should be short. For example*, *not be more than 5 minutes “.* Obstetrician II.

Other respondents were worried about having a very short video which could result in insufficient information given.

*“If the video is very fast and short, people may miss out on some information. So, I suggest the video to be about 10 minutes long”.* FGD lady aged_15-17yrs.

*“It depends on how the information is given in the video. It needs actors who talk fast in about three to four minutes. Because, if it lasts longer, one can walk away before the video ends”.* FGD lady aged_15-17yrs.

#### Engaging or edutaining video

Respondents emphasized that the video should be able engage women. This can be achieved by making the video edutaining.

“*…. but I suggest the developed video to be edutaining. Because people are good at watching videos if the videos are entertaining. So, it should not be a boring video”.* MoH official I.

#### Use of real human beings to act in the video

Respondents suggested that the video should be designed in the form of a play to attract attention and concentration.

*“A play would work better, but for talking, some of our speeches bore. It needs something that can attract attention. People can be very attentive to watch a play”* Midwife. II.

Despite mixed feelings associated with the use of real human beings or animations like cartoons in the video, most of the respondents preferred real human beings because it could provide an impression of reality and seriousness. Moreover, respondents preferred having an individual acting other than providing a narrative story.

*“I think having someone act is the best because it attracts someone’s attention to watch how the story goes. But narrating at times may be boring. Some people don’t take cartoons seriously, they think cartoons or animations are for children. But if it is a human talking and identifying themselves, people usually take it seriously “.* Obstetrician I.

*“…a video should use a person who can explain to the women and understand. That person can explain the benefits of swallowing the medicine for malaria prevention, and the consequences of not swallowing the medicines. And that person should be serious to show what happens if you swallow or not swallow the medicine”.* FGD lady 18-24yrs.

#### Messaging or providing information in the video

When asked about the key messages that can be included or provided in the video, respondents mentioned that the information should include the benefits of taking and risks of not taking malaria preventive therapy in pregnancy, and instructions of taking the therapy.

#### Including information on the benefits of taking and the risks of not taking malaria preventive therapy in pregnancy

Respondents mentioned that the video should have information emphasizing the purpose and benefits of malaria preventive therapy in pregnancy, and the risks of not taking the therapy.

*“The video should show the real importance of taking these medicines because we always communicate the risks of not taking the medicine and forget to emphasize the purpose and benefits”.* midwife I.

However, respondents mentioned that the purpose and benefits should be more emphasized than risks of not taking the therapy. This is because over emphasizing the potential risks of not taking IPTp-SP or co-trimoxazole can cause fear in women and deter them from following instructions. Additionally, over focusing on the potential risks of not taking malaria preventive therapy can sometimes be interpreted as coercion or forcing women to take the drugs. Therefore, the video should be directed towards motivating women to take the drugs.

*“….and include information that motivates or encourages them to take the drugs. For example, giving them assurance about protection of both the woman and the unborn baby, and being healthier for both the woman and the unborn baby”, Obstetrician II.* However, some respondents recommended that adequate information on the potential risks of not taking the drugs should be included as well.

*“We can also include adverse effects of malaria in pregnancy such as severe anemia, miscarriage, fetal death, and low birth weight”*, midwife IV.

*“Let us also include problems like getting miscarriages, babies dying in womb, and mother herself dying. If a woman hears such problems, she can say; let me go and get drugs so that I don’t get problems”.* FGD_18–24 years.

#### Including information on the instructions of taking malaria preventive therapy

Respondents mentioned that the instructions and schedules of taking these medicines should be included in the video.

*“The video should include the drugs to use, how to use them, and their benefits, and then the effects of not taking the drugs”.* FGD woman aged _25-49years.

### Delivery of the message of malaria preventive therapy in the video

Respondents proposed practices that can be used in delivering the developed video to the target population. They mentioned the appropriate stage when pregnant women should start watching the video, the appropriate time of watching the video, channels of watching the video, and places where to watch the video from.

#### The stage at which a pregnant woman can start watching the video

Many of the respondents recommended watching such educational videos right from preconception or early pregnancy and through the entire pregnancy period.

*“These women should be educated about malaria preventive therapy right before they become pregnant (preconception). And the information should be given to them continuously on a regular basis throughout pregnancy”*, midwife III.

*“As early as we get in touch with the pregnant woman, we should provide them with malaria prevention messages. Even if someone has not reached the time of starting IPTp-SP, they can be looking forward to that time. And remember for co-trimoxazole, it should be started as soon as one becomes pregnant”.* Obstetrician I.

#### The appropriate time, channels, and places of watching the video

Respondents also suggested various channels through which the video should be delivered. Respondents mentioned watching the video on television screens during antenatal care clinics or at home and watching the video on phones (smart phones). However, respondents emphasized that the video should be aired out at all points where pregnant women are found especially the ANC and reproductive health clinics.

*“Using media like the televisions, this is the information someone can watch for example before the news or favorable programs, for example, women like soaps. You know that we have a bigger audience before the news, before the soaps and on peak of some programs”.* Obstetrician II.

There were no noticeable differences regarding preferences to watch the video between women living with HIV and those without.

*“I prefer watching the video on television screen at home because when I come to the health facility, I can be busy looking for health workers to work on me. I may not concentrate to watch the video. But when I am at home, I can even rewind the video several times so that I get to understand the message”.* FGD lady aged_18–24 years.

*…. “You realize that someone can sit somewhere pick up their phone and watch the videos that they have on their phones. And if there is a TV, for example in the clinic or at home, people will watch it.* MoH official II.

## Discussion

In this study, we aimed to obtain client and stakeholder opinions, attitudes, and perceptions to develop and design an educative video to improve knowledge and uptake of malaria preventive therapy in pregnancy. This study revealed that strategies and actions are needed to improve knowledge of malaria preventive therapy among pregnant women such as engaging of male sexual partners or treatment supporters and providing effective regular health education talks; pregnant women and key stakeholders were positive towards developing and designing a video-based intervention to educate women on malaria preventive therapy in pregnancy; a need for proper packaging and framing of the message of malaria preventive therapy in the video such as having a video that is short, edutaining, and focused on both benefits of taking and risks of not taking the malaria preventive therapy in pregnancy; and a need for effective means of delivering the video to pregnant women such as use of television sets to display the video at the clinic or at home, use of smart phones and social media, and ensuring that pregnant women access or watch the video as soon as possible after conception. The implications of these findings are as below.

### Strategies and actions are needed to improve knowledge of malaria preventive therapy among pregnant women

The current study suggests a need for engaging male sexual partners and treatment supporters when educating women about malaria preventive therapy. This is because these can support the women in understanding the purpose and the benefits of the therapy. The male partner and treatment supporter can also remind the women to take their medicine and, hence increasing adherence. Previous studies have also proposed this strategy [43, 44]. It’s reported that male partner involvement in ANC services is associated with better utilization of maternal health service, decreased delays in making the decision to seek medical attention, improves material, emotion, and physical support, and increased likelihood of adherence to medical care advice [45, 46]. Therefore, more efforts are needed to involve male partners or treatment supporters in maternal and child health services.

Provision of effective regular health education talks. The study revealed that there is a need for more education talks and these talks should be effective. Many women don’t understand the purpose and the benefits of taking malaria preventive therapy, and don’t understand the associated risks of not taking the therapy. Other women may forget what they learnt previously, and policies and guidelines change overtime. Therefore, there is a need to have regular education talks concerning malaria preventive therapy during pregnancy. The importance of regular education talks to pregnant women has been reported previously [47, 48]. However, there is a need to ensure that these talks are effective for all categories of pregnant women. In LRS like Uganda, education talks may be ineffective due to low ratio of health workers to patients [28]. This study revealed that the low health worker to patient ratio results in inadequate sensitization and education of pregnant women on malaria preventive therapy during ANC visits; and therefore, more scalable, and cost-effective interventions may be needed.

### Positive reactions towards developing and designing a video-based intervention to educate women on malaria preventive therapy in pregnancy

This study revealed that pregnant women and key stakeholders were positive about the video based educational strategy in increasing knowledge and uptake of malaria preventive therapy in pregnancy. The strategy was seen as an innovation in the field of maternal and child health services. This finding was consistent with a study done in Ghana which showed that video job-aids were welcomed and effectively supported the delivery of seasonal malaria chemoprevention [49]. In this Ghanaian study, the conveyed videos enhanced learning and information retention. The videos also reinforced messages due to the fact that they could be viewed at any time and repeatedly [49]. Kim et al. also reported that brief video-based educational interventions are promising approaches in improving health related knowledge among the target population [29].

### Proper packaging and framing of the message of malaria preventive therapy in the video

The current study emphasized a short, concise, and focused educative video. The study in Ghana also utilized short videos, which were found to be easily distributed on social media and thus reached many people in a short period of time [49]. Therefore, the current study showed that a short video of about 5 min maybe appropriate to improve knowledge and uptake of malaria preventive therapy in pregnancy.

Regarding the contents of the video, the current study showed that the video should emphasize the purpose and benefits of malaria preventive therapy in pregnancy and be edutaining. This was similar to the study conducted in Guinea which showed that positive messaging of the video content is a consensus of good practice and successful video-based education as reflected in the WHO and national guidelines on malaria prevention [50, 51]. However, the current study also revealed that the risks of not taking malaria preventive therapy should equally be emphasized. It was mentioned that pregnant should also be informed about the adverse effect of malaria in pregnancy including miscarriage, low birth weight babies, and maternal or fetal death. A study among diabetic patients showed that patients who received negatively framed message showed significantly more favorable attitudes and perceived control toward diabetes self-care than those who viewed the positively framed message [52]. Therefore, the video may have both the information about the benefits of taking malaria preventive therapy and information about the risks of not taking the therapy.

The study revealed that the video should consistently and address concerns relevant to malaria etiology and prevention measures. This was similar to the recommendations from the Ugandan national health survey [53]. Relatedly, studies have also shown that highlighting and identifying the correct health messages in a consistent and repetitive manner boosts audience knowledge and malaria intervention uptake [49, 54].

### Delivering the video-based information to pregnant women

The study revealed that all women of reproductive age should be a target for this video-based education. This was consistent with previous reports and World Health Organization recommendations [55, 56]. However, the findings of the current study emphasized women to receive this video-based education as soon as they become pregnant and throughout pregnancy. This was because malaria preventive medication like cotrimoxazole needs to be taken as soon as one becomes pregnant and throughout the pregnancy. Additionally, all women should know what they are supposed to do such as taking IPTp-SP even if they are not yet in 2nd trimester. This strategy has been previously recommended [57, 58]. Therefore, women may be provided with the video-based education for malaria prevention as soon as they conceive.

The study revealed that the video-based education may be provided on television sets in maternal and childcare clinics as well as sexual and reproductive health clinics. A previous study showed that planning health messaging and delivery systematically offers the chance to fulfill people’s actual informational demands in addition to creating effective communication tactics [59]. It was also revealed that the video can also be viewed at home on a television set or anywhere on smart phone. A systematic review showed that short video education messages can be repeatedly watched from anywhere [49]. Therefore, the educative video for malaria preventive therapy in pregnancy may be designed to be viewed on television sets at clinics and home and on smart phones.

## Strengths and limitations

Our study strengths include using iterative client centered processes to tailor and develop a video to improve knowledge on malaria prevention therapy among pregnant women. The study obtained data through a participatory process from pregnant women and key stakeholders in maternal and child health which ensured triangulation and complementation of information from the respondents. The study included HIV negative and HIV-positive women as well as women of different age groups, which enabled us to obtain varied responses and representation from various categories of pregnant women. Our study was conducted in an urban setting therefore, the findings are limited to the urban population and may not be generalized to rural communities.

## Conclusions

In conclusion, pregnant women, health care workers, and ministry of health officials were enthusiastic and positive about developing and designing an educative video for malaria preventive therapy during pregnancy. However, they suggested a short edutaining video with properly packaged and framed information focused on both benefits of taking and risks of not taking the malaria preventive therapy in pregnancy. They also suggested delivering the video to pregnant women as soon as they conceive through television sets at maternal and reproductive health clinics and through smart phones. Data from this study was used to develop and design and educative video for malaria preventive therapy in pregnancy.

### Electronic supplementary material

Below is the link to the electronic supplementary material.

Supplementary Material 1

## Data Availability

The dataset used and analyzed during this study is available from the corresponding author on reasonable request.

## Abbreviations

AIDS: Acquired immunodeficiency syndrome
FGD: Focus Group Discussion
HIV: Human Immunodeficiency Virus
IDI: In-Depth Interviews

## References

[1] World Health Organization. A strategic framework for malaria prevention and control during pregnancy in the African region. World Health Organization. Regional Office for Africa; 2004.

[2] World Health Organization. *World Malaria Report 2016 (World Health Organization, Geneva)* 2016.

[3] Ministry of Health. U.N.M.C.D., *Malaria indicator survey*, T.D.P.I. Uganda Bureau of Statistics, Editor. 2018–29.

[4] Arinaitwe E (2013). Intermittent preventive therapy with sulfadoxine-pyrimethamine for malaria in pregnancy: a cross-sectional study from Tororo. Uganda PloS One.

[5] Saito M (2020). Deleterious effects of malaria in pregnancy on the developing fetus: a review on prevention and treatment with antimalarial drugs. Lancet Child Adolesc Health.

[6] Twabi HS, Manda SO, Small DS (2020). Assessing the effects of maternal HIV infection on pregnancy outcomes using cross-sectional data in Malawi. BMC Public Health.

[7] Enuma JN (2022). Malaria an opportunistic infection in HIV/AIDS patients?-A Nigerian experience. Afr J Lab Med.

[8] Flateau C, Le Loup G, Pialoux G (2011). Consequences of HIV infection on malaria and therapeutic implications: a systematic review. Lancet Infect Dis.

[9] Vashishtha VM (2008). World Malaria Report 2008: a billion-dollar moment for a centuries old disease. Indian Pediatr.

[10] World Health Organization, World malaria report 2020: 20 years of global progress and challenges. World malaria report 2020: 20 years of global progress and challenges. 2020: p. 299.

[11] World Health Organization. HIV treatment and care: WHO HIV policy adoption and implementation status in countries: factsheet. World Health Organization; 2019.

[12] UNAIDS, t.J.U.N.P.o.H.A. *IN DANGER: UNAIDS Global AIDS Update 2022. Geneva: Joint United Nations Programme on HIV/AIDS; 2022. Licence: CC BY-NC-SA 3.0 IGO*. 2022.

[13] Mangusho C (2023). High prevalence of malaria in pregnancy among women attending antenatal care at a large referral hospital in northwestern Uganda: a cross-sectional study. PLoS ONE.

[14] Braun V (2015). Lack of effect of intermittent preventive treatment for malaria in pregnancy and intense drug resistance in western Uganda. Malar J.

[15] Okiring J (2019). Household and maternal risk factors for malaria in pregnancy in a highly endemic area of Uganda: a prospective cohort study. Malar J.

[16] UGANDA AIDS COMMISSION SECRETARIAT. U. Factsheet - Facts on HIV and AIDS in Uganda 2021 (Based on Data ending 31st December 2020). Uganda AIDS commission 2021; https://uac.go.ug/media/attachments/2021/09/13/final-2021-hiv-aids-factsheet.pdf.

[17] Gasasira AF (2010). Effect of trimethoprim-sulphamethoxazole on the risk of malaria in HIV-infected Ugandan children living in an area of widespread antifolate resistance. Malar J.

[18] Mermin J (2006). Effect of co-trimoxazole prophylaxis, antiretroviral therapy, and insecticide-treated bednets on the frequency of malaria in HIV-1-infected adults in Uganda: a prospective cohort study. Lancet.

[19] Fokam EB (2016). Assessment of the usage and effectiveness of intermittent preventive treatment and insecticide-treated nets on the indicators of malaria among pregnant women attending antenatal care in the Buea Health District, Cameroon. Malar J.

[20] World Health Organization. Guidelines for the treatment of malaria, World Health Organization. Geneva, Switzerland.[Google Scholar]; 2015.

[21] Al Khaja KA, Sequeira RP (2021). Drug treatment and prevention of malaria in pregnancy: a critical review of the guidelines. Malar J.

[22] Ministry of Health. U., *Consolidated guidelines for Prevention and Treatment of HIV and AIDs in Uganda February 2020* 2020.

[23] Nekaka R (2020). Malaria preventive practices and delivery outcomes: a cross-sectional study of parturient women in a tertiary hospital in Eastern Uganda. PLoS ONE.

[24] Okethwangu D (2019). Factors associated with uptake of optimal doses of intermittent preventive treatment for malaria among pregnant women in Uganda: analysis of data from the Uganda Demographic and Health Survey, 2016. Malar J.

[25] Rassi C (2016). Assessing supply-side barriers to uptake of intermittent preventive treatment for malaria in pregnancy: a qualitative study and document and record review in two regions of Uganda. Malar J.

[26] Mutulei ACNe (2013). Factors influencing the uptake of intermittent preventive treatment for malaria in pregnancy: evidence from Bungoma East District, Kenya. Am J Public Health Res.

[27] Wanzira H (2016). The challenge of using intermittent preventive therapy with sulfadoxine/pyrimethamine among pregnant women in Uganda. Malar J.

[28] Bolan N (2021). Human resources for health-related challenges to ensuring quality newborn care in low-and middle-income countries: a scoping review. Global Health: Sci Pract.

[29] Kim MH (2020). The video intervention to Inspire Treatment Adherence for Life (VITAL start): protocol for a multisite randomized controlled trial of a brief video-based intervention to improve antiretroviral adherence and retention among HIV-infected pregnant women in Malawi. Trials.

[30] Sweat M, O’Donnell C, O’Donnell L (2001). Cost-effectiveness of a brief video-based HIV intervention for African American and latino sexually transmitted disease clinic clients. Aids.

[31] Fisher HH (2011). Evaluation of an HIV prevention intervention for African americans and hispanics: findings from the VOICES/VOCES Community-based Organization Behavioral Outcomes Project. AIDS Behav.

[32] Wong IY (2006). Development and assessment of an innovative culturally sensitive educational videotape to improve adherence to highly active antiretroviral therapy in Soweto, South Africa. JAIDS J Acquir Immune Defic Syndr.

[33] Tuong W, Larsen ER, Armstrong AW (2014). Videos to influence: a systematic review of effectiveness of video-based education in modifying health behaviors. J Behav Med.

[34] Statistics UBo. *The National Population and Housing Census 2014– Main Report, Kampala, Uganda*. 2016.

[35] UBOS. *UBOS Uganda demographic and health survey 2016: key indicators report. Kampala, Uganda (2017)* 2017.

[36] Ministry of Health, U. *Annual health sector performamnce report, financial 2017/2018*. 2018.

[37] Walker JL. Research column. The Use of Saturation in qualitative research. Can J Cardiovasc Nurs, 2012. 22(2). 22803288

[38] Booth A et al. *COREQ (consolidated criteria for reporting qualitative studies)* Guidelines for reporting health research: A user’s manual, 2014: pp. 214–226.

[39] Creswell JW, Creswell JD. Research design: qualitative, quantitative, and mixed methods approaches. Sage; 2017.

[40] Strecher VJ, Rosenstock IM (1997). The health belief model. Camb Handb Psychol Health Med.

[41] Thorne S (2008). Interpretive description (Vol. 2). Walnut Creek.

[42] Nan X, Daily K, Qin Y (2018). Relative persuasiveness of gain-vs. loss-framed messages: a review of theoretical perspectives and developing an integrative framework. Rev Communication.

[43] Alemi S (2021). Male participation in antenatal care and its influence on their pregnant partners’ reproductive health care utilization: insight from the 2015 Afghanistan demographic and Health Survey. J Biosoc Sci.

[44] Omolola A (2022). Male participation in Antenatal Care and its effect on pregnancy. J Health Med Nurs.

[45] World Health Organization. *Engaging men, addressing harmful masculinities to improve sexual and reproductive health and rights* 2019.

[46] Tokhi M (2018). Involving men to improve maternal and newborn health: a systematic review of the effectiveness of interventions. PLoS ONE.

[47] Jibril UN (2018). Health education intervention on knowledge and accessibility of pregnant women to antenatal care services in Edu, Kwara State, Nigeria. Int J Womens Health Reprod Sci.

[48] Raoof AM, Al-Hadithi TS (2011). Antenatal care in Erbil city-Iraq: assessment of information, education and communication strategy. DMJ.

[49] Owusu-Addo E, Owusu-Addo SB. *Effectiveness of health education in community-based malaria prevention and control interventions in sub-Saharan Africa: a systematic review* 2014.

[50] Scott S (2022). The use of video job-aids to improve the quality of seasonal malaria chemoprevention delivery. PLOS Digit Health.

[51] Mutanyi JA, et al. Determinants of the uptake of intermittent preventive treatment of malaria in pregnancy with sulphadoxine pyrimethamine in Sabatia Sub County, Western Kenya. Volume 10. Infectious Diseases of Poverty; 2021. p. 106. 1. 10.1186/s40249-021-00887-4 PMC8343925 34362443

[52] Park J, Kim SH, Kim JG (2020). Effects of message framing and health literacy on intention to perform diabetes self-care: a randomized controlled trial. Diabetes Res Clin Pract.

[53] Ameyaw EK (2022). Uptake of intermittent preventive treatment of malaria in pregnancy using sulfadoxine-pyrimethamine (IPTp-SP) in Uganda: a national survey. Malar J.

[54] Darteh EKM (2021). Factors influencing the uptake of intermittent preventive treatment among pregnant women in sub-saharan Africa: a multilevel analysis. Archives Public Health.

[55] Kilfoyle KA (2016). Health literacy and women’s reproductive health: a systematic review. J Women’s Health.

[56] Organization WH. *WHO recommendations on adolescent sexual and reproductive health and rights* 2018.

[57] Wylie B (2020). Malaria in pregnancy: prevention and treatment.

[58] Almalik MM, Mosleh SM (2017). Pregnant women: what do they need to know during pregnancy? A descriptive study. Women Birth.

[59] World Health Organization. *WHO Strategic Communications Framework for Effective Communication*. 2017.

