# Supplementary material for: Attitudes and perceptions towards developing a health educational video to enhance optimal uptake of malaria preventive therapy among pregnant women in Uganda: a qualitative study involving pregnant women, health workers, and Ministry of health officials
Source: BMC Health Serv Res. 2024 Apr 18;24:484. doi: 10.1186/s12913-024-10944-x (PMC11027371; doi:10.1186/s12913-024-10944-x)
Supplement: Supplementary file 1 — Supplementary Material 1 [file 12913_2024_10944_MOESM1_ESM.pdf]

Participant ID Sticker

MU-JHU CARE LIMITED
MU-JHU Research Collaboration
Makerere University, Kampala
The Johns Hopkins University, Baltimore
P. O Box 23491, Kampala, Uganda
Telephone: +256-414-541044 Facsimile: +256-414-543002

PARTICIPANT FOCUS GROUP DISCUSSION GUIDE

**Protocol Title:**	A novel video-based intervention to enhance optimal uptake of malaria preventive therapy: a pilot study of a health educational approach to malaria prevention during pregnancy
**Short Title**:	PREvention of malaria in pregnancy using Video-based Education to eNhance opTimal malaria preventive therapy knowledge and uptake (PreVent) Study
**Funding Agency:**	Division of AIDS, US National Institute of Allergy and Infectious Diseases, US Eunice Kennedy Shriver National Institute of Child Health and Human, Development, US National Institute of Mental Health, US National Institutes of Health.
**Protocol No:**	
**Principal Investigator**:	Rita Nakalega
**Telephone Number:**	0414-541044
**Date:**	15 Nov 2021

INSTRUCTIONS for the Facilitator: How to use the FGD Guide

1. There are two levels of questions:

    - Primary discussion questions: appear in **bold** text and represent all the topics you will need to cover by the end of the interview. The questions are written to ensure some consistency across FGDs but you are not required to read them verbatim. You may adapt the questions and/or ask them in a different order, depending on how the discussion develops.
    - Probing topics: are indicated with a bullet. If you find that the participants provide little information in response to the primary question, these probing topics may be used to encourage further discussion. You are not required to cover every topic listed.

2. *Instructions/suggestions to facilitator are in italics and [brackets].*

3. The FGD guide is not meant to be used to take notes. Rather, you should use the separate notes form, where you will also insert your initials, the PTIDs of all participants, as well as the date, start and end time of the discussion.

Before starting the FGD, ensure that all participants have provided written informed consent.

A. Introduction
[*Facilitator should explain the following points – PLEASE DO NOT READ VERBATIM:*]<br><br>• Purpose of FGD:<br>o Generate information that can be included in the PreVent intervention video.<br>o Generate ideas on how the PreVent intervention video can be displayed to the pregnant women<br>• Affirm to the participants that they are the experts and that all answers are valid: no right and wrong<br>• Invite differing opinions<br>• Remind participants that the discussion is confidential, so personal information won't be shared outside of the study<br>• Tell participants to use pseudonyms for themselves and anyone else they mention so as to preserve confidentiality<br>• Participants should identify themselves with their pseudonym each time prior to raising a point and speak one at a time so that the audio recorder can capture everything<br>• Remind participants to keep cellphones silent throughout discussion to avoid disruption of audio recording<br>• *[Turn audio recorder on]* Ask participants to confirm for audio recorder that they agree to participate in FGD *[be sure to get a verbal okay from all members before continuing]*
B. The risk of malaria in pregnancy

Purpose: To understand the perceived risk of Malaria in pregnancy among pregnant women.

1. **Tell me what you know about malaria in pregnancy**

<u>*Possible probing topics:*</u>

- KEY PROBE: How common is malaria in pregnancy?
- KEY PROBE: How does malaria in pregnancy affect the pregnant woman?
- KEY PROBE: How does malaria affect the unborn baby?
- KEY PROBE: What makes pregnant women fail to understand the danger of malaria in pregnancy
- KEY PROBE: What can make pregnant women understand the danger of malaria in pregnancy

C. Malaria preventive therapy in pregnancy

Purpose: To understand if pregnant women the available malaria preventive therapies in pregnancy.

2. **Tell me what you know about malaria prevention therapy in pregnancy?**

<u>*Possible probing topics:*</u>

- *KEY PROBE*: What medication are taken in pregnancy to prevent malaria, and how often is it supposed to be taken?
- *KEY PROBE*: Tell me what hinders uptake of malaria preventive therapy in pregnancy
- KEY PROBE: What can be done to increase uptake of malaria preventive therapy in pregnancy

D. Video-based educative malaria prevention message

3. **What do you think can be included in the PreVent video message?**

<u>*Possible probing topics:*</u>

- *KEY PROBE*: What message can easily remind you taking malaria preventive therapy in pregnancy?
- *KEY PROBE*: Where do easily think can watch this video from? home, health facility or both?
- *KEY PROBE*: Which gadget is easy for you to watch this video? TV, phone, tablet or both?

Wrap Up

4. **Thank you for taking the time to share your opinions with us today. We truly appreciate your willingness to participate and discuss your thoughts and ideas with us. We've now reached the end of our discussion. Do you have any additional comments about PreVent video experiences?**
